# Comparison of MF and RMS EMG parameters in women with TMD and asymptomatic women during sustained bilateral submaximal bite force protocol

**DOI:** 10.3389/fpain.2026.1962220

**Published:** 2026-10-08

**Authors:** Paulo Fernandes Pires, Elisa Bizetti Pelai, Samara Caroline Fernandes Galvani, Bianca Pulino, Marcio de Moraes, Ester Moreira de Castro-Carletti, Fabiana Foltran-Mescollott, Raphael Capelli Guerra, Delaine Rodrigues-Bigaton

**Affiliations:** 1 Graduate Program in Physical Therapy, State University of Northern Paraná – UENP – Jacarezinho (PR), Jacarezinho, Brazil; 2 Post-Graduate Program in Oral and Maxillofacial Surgery, Piracicaba Dental School, State University of Campinas – UNICAMP, Piracicaba, Brazil; 3 Hospital Sírio-Libanês, Teaching and Research Institute, São Paulo, Brazil; 4 Albert Einstein College of Health Sciences, São Paulo, Brazil; 5 Department of Oral Diagnosis, Oral and Maxillofacial Surgery Division, Piracicaba Dental School, State University of Campinas – UNICAMP, Piracicaba, São Paulo, Brazil; 6 Post-Graduate Program in Human Movement Sciences, Methodist University of Piracicaba – UNIMEP – Piracicaba (SP), Brazil; 7 Post-Graduate Program in Oral and Dental Biology, Piracicaba Dental School, State University of Campinas – UNICAMP – Piracicaba (SP), Brazil

**Keywords:** bite force, diagnosis, electromyography, facial pain, temporomandibular joint disorders

## Abstract

**Introduction:**

Muscle fatigue is characterized by a reduction in the muscle's ability to generate force during voluntary contractions and can be assessed using surface electromyography (EMG) through the parameters of median frequency (MF) and root mean square (RMS).

**Objective:**

This study aimed to compare the Median Frequency (MF) and Root Mean Square (RMS) values of the masseter and anterior temporalis muscles between women diagnosed with myogenic Temporomandibular Disorder (TMD) and asymptomatic women during a sustained submaximal bilateral bite task on the molars.

**Methods:**

This cross-sectional study included 86 women, distributed into TMD and Control groups based on DC/TMD diagnosis. The angulation coefficient (*β*) of the linear regression line of the RMS and MF was evaluated in the submaximal bilateral molar bite task at 50% of the maximum bite force for 30 s.

**Results:**

A negative intragroup *β* coefficient was observed for MF in all muscles in both groups (*p* < 0.001 for all), indicating a decline in MF over time. A positive intragroup *β* coefficient was observed for RMS in all muscles in both groups, indicating an increase in RMS over time. The Control Group showed a significantly greater negative *β* coefficient for MF in the right masseter (*p* = 0.01) and left masseter (*p* = 0.01) muscles, indicating a steeper decline in MF during the fatigue protocol, and a significantly greater positive *β* coefficient for RMS in the left anterior temporalis muscle (*p* = 0.01), indicating a steeper increase in RMS, compared to the TMD Group.

**Conclusion:**

The long-duration submaximal bite task produced signs of muscle fatigue in both groups; however, signs of fatigue were significantly more pronounced in the Control Group, specifically in the masseter muscles (MF decline) and the left anterior temporalis muscle (RMS increase), challenging our initial hypothesis of greater fatigue in the myogenic TMD Group.

## Introduction

Muscle fatigue is defined as a decline in the capacity of a muscle to generate force or power during voluntary contractions. This process may involve peripheral mechanisms (occurring within the muscle tissue or at the neuromuscular junction) or central mechanisms (originating in the brain or spinal cord) (1, 2).

Surface electromyography (EMG) is the predominant tool used in the evaluation of muscle fatigue protocols during bite tasks in individuals with temporomandibular disorders (TMD) and asymptomatic individuals (3–7).

Muscle fatigue is commonly assessed by analyzing the EMG signal in the frequency domain, using parameters such as median frequency (MF EMG) or the mean frequency index derived from the power spectral density. During fatigue, these frequency parameters shift toward lower values, reflecting a decrease in the conduction velocity of muscle fibers. The myoelectric signal in the amplitude domain can also be analyzed using the parameters of root mean square EMG (RMS EMG) or the integral of the EMG signal (IEMG). During fatigue, an increase is expected in the amplitude of RMS and a decrease in the MF, mainly due to the additional recruitment of motor units to maintain the required strength level during the activity in execution (7).

De Luca (1997) suggests evaluating fatigue using muscle contractions with a strength level higher than 30% of the maximum strength, triggering the occlusion of the blood flow from the contracting muscle and helping to limit the interference of extra-muscular factors during sustained contraction. In this way, fatigue protocols can be performed with sub-maximal protocols (8).

Some studies (10, 11) have evaluated the muscle fatigue parameter between individuals with TMD and asymptomatic individuals using a short fatigue protocol (5 to 10 s) during a maximum isometric bite directly on the occlusal surface of the teeth and observed significantly lower MF EMG values in the masticatory muscles of individuals with TMD, as well as a significant reduction in the MF EMG values of the masticatory muscles from the beginning to the end of the task in individuals with TMD compared to asymptomatic individuals.

Xu et al. (2017) (7) and Pitta et al. (2015) (5) compared short and long fatigue protocols and found significant increases in RMS EMG of the anterior temporalis muscle from the beginning to the end of the task in the asymptomatic group, as well as significant reduction in MF EMG values in both groups, with no significant differences between TMD and asymptomatic groups.

Considering that several studies have used short protocols with maximum isometric contraction or unilateral bite, and the findings were inconclusive, the present study can be justified by the gap in the literature regarding the use of a long submaximal protocol with a bilateral bite dynamometer to compare individuals with TMD and asymptomatic individuals. Our hypothesis was based on the premise that the TMD Group, already presenting with muscle dysfunction, would have a reduced capacity to maintain force, resulting in earlier and more pronounced signs of fatigue. However, we also acknowledge that previous studies have reported conflicting results between TMD and asymptomatic groups, which underscores the need for the present investigation.

The present study therefore aimed to compare the electromyographic parameters (MF and RMS) of the masseter and anterior temporalis muscles during a long-duration (30-second) fatigue protocol involving a sustained bilateral submaximal molar bite task in women with myogenic TMD and asymptomatic women. A bilateral bite dynamometer was used to monitor and maintain the target submaximal bite force (50% of maximum bite force) throughout the task. This design addresses the gap in the literature regarding the use of long-duration bilateral submaximal protocols for evaluating muscle fatigue in TMD populations.

## Materials and methods

### Study design

This is a cross-sectional study, in accordance with the Declaration of Helsinki. The protocol was approved by the local Research Ethics Committee of Piracicaba Dental School, Universidade Estadual de Campinas - UNICAMP (approval protocol no. 25/2015). Written informed consent was obtained from all individual participants included in the study. Participants were informed about the study procedures, and all provided their freely-given, informed consent before enrollment.

### Subjects

The final sample of this research was composed of women, divided into two groups: myogenic TMD group (with possible intra-articular comorbidities) and control group. Both TMD and Control groups were recruited from the same source population attending the Surgery Sector of a School of Dentistry in Piracicaba, São Paulo, Brazil. Recruitment and data collection occurred between January 2016 and December 2018**.** The current sample represents an independent cohort of participants and is not overlapping with the sample reported in our previous study (Pires et al., 2023), although both studies recruited from the same population and applied similar inclusion criteria. The present sample was recruited at a different period and consists of distinct individuals.

### Inclusion and exclusion criteria

Women aged between 18 and 40 years with a Body Mass Index (BMI) < 25 kg/m^2^ were selected for the study. In addition, for the TMD group, the volunteers were required to have a primary diagnosis of myogenic TMD according to the Diagnostic Criteria of Temporomandibular Disorders (DC/TMD) (9) (myalgia and/or myofascial pain), who may also present with comorbid intra-articular diagnoses such as arthralgia or disc displacement, as detailed in Table 1, and present pain and/or fatigue in the masticatory muscles during functional activities for more than six months, while for the control group, the volunteers were required not to present pain and/or any diagnosis of TMD according to the DC/TMD.

**Table 1:** Diagnosis of all volunteers (TMD and control group) according to DC/TMD. Data are expressed as frequency (*n* = 86).

Diagnosis	Control Group (*n* = 43)	TMD Group (*n* = 43)
Myalgia	-	22
Myofascial pain	-	15
Myofascial pain with referral	-	6
Disc displacement with reduction (R/L)	-	14 (10/9)
Disc displacement without reduction, with limited opening (R/L)	-	4 (4/4)
Arthralgia (R/L)	-	34 (25/28)

DC/TMD, Diagnostic Criteria of Temporomandibular Disorder; R/L, Right/Left.

Women who received physical or pharmacological treatment (analgesic, anti-inflammatory, and muscle relaxant), with dental losses, who used total or partial dentures, with a history of trauma on the face and TMJ, a history of subluxation or dislocation of the TMJ, and diagnosed with degenerative joint diseases through DC/TMD were excluded from the study.

### Sample size calculation

A sample size calculation was performed based on a pilot study with 5 volunteers per group. The outcome used was the RMS EMG value of the anterior temporalis muscle during the bite phase, as this was the parameter for which pilot data were available at the time of planning. The mean and standard deviation values of RMS for the Control Group and TMD Group were, respectively, 0.16 ± 0.06% and 0.12 ± 0.06%, yielding an effect size (Cohen's d) of 0.67**.** Setting a power of 80% and a 5% alpha level, the sample size was determined as 36 volunteers per group. To account for potential losses, we recruited 43 volunteers per group using GPower® software, version 3.1.9.2.

The final sample was composed of 86 women, divided into two groups: myogenic TMD group, formed by 43 women, and control group, formed by 43 women, according to the flowchart shown in Figure 1.

**Figure 1:**
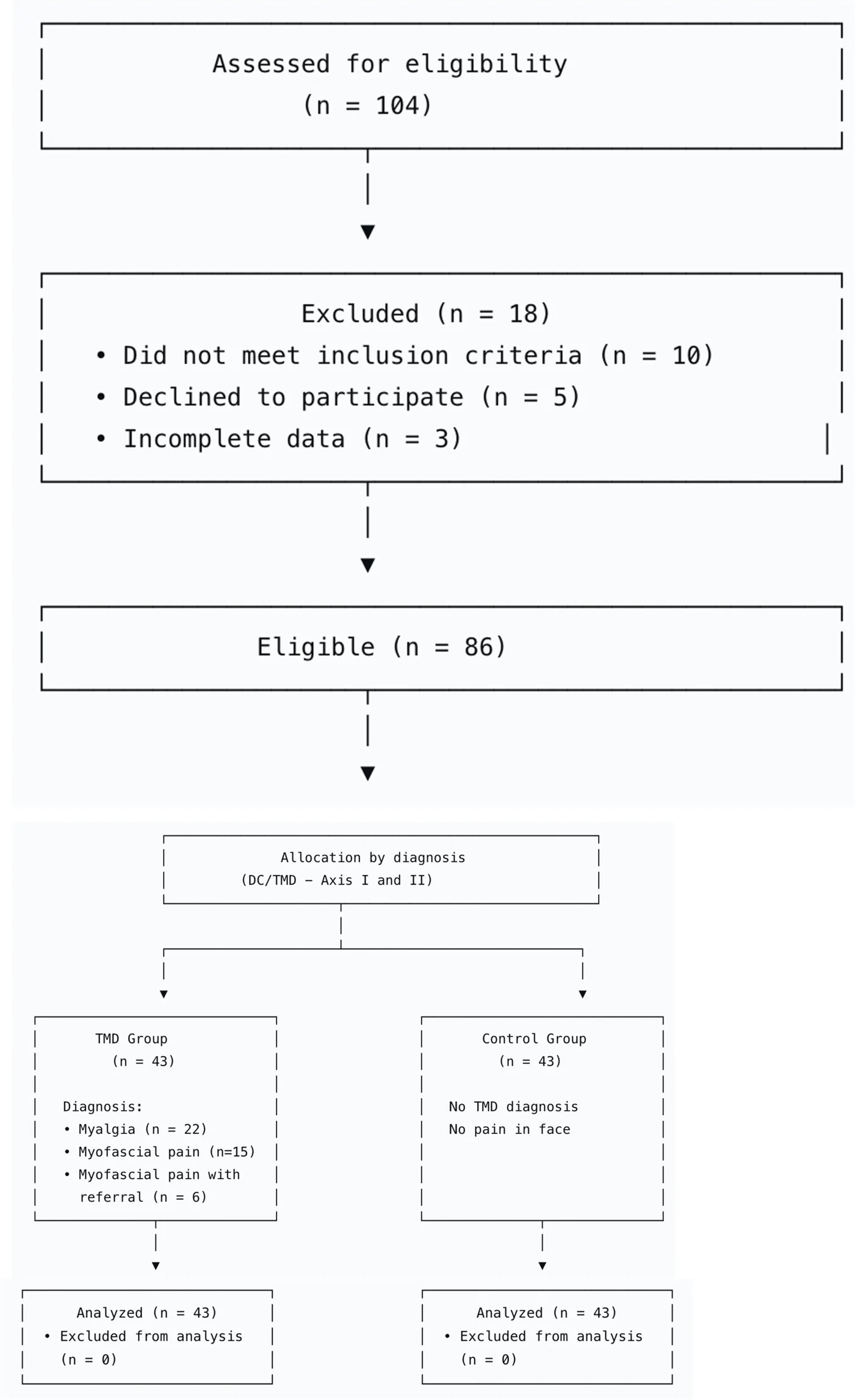
Flowchart of the volunteers recruitment. Participant flow diagram according to STROBE guidelines for cross-sectional studies. A total of 104 individuals were assessed for eligibility. Of these, 18 were excluded: 10 did not meet the inclusion criteria (e.g., BMI ≥ 25 kg/m^2^, previous treatment for TMD, dental losses), 5 declined to participate, and 3 had incomplete data. The remaining 86 participants were allocated to the TMD Group (*n* = 43) or Control Group (*n* = 43) based on DC/TMD diagnosis. All 43 participants in each group were included in the final analysis, with no exclusions after data collection. TMD, Temporomandibular Disorder; DC/TMD, Diagnostic Criteria for Temporomandibular Disorders.

### Evaluation

#### Diagnostic criteria of temporomandibular disorder (DC/TMD)

The DC/TMD contains two scales: Axis I (painful TMD, intra-articular disorders, degenerative joint disease, and joint subluxation) and Axis II (pain-related impairment of daily activities, depression, and non-specific somatic symptoms) (12). Schiffman et al. (9) validated the assessment of TMD by the DC/TMD, using questionnaires and physical tests. Their results, although sometimes lacking a gold standard, led to the selection of valid diagnostic criteria for differentiating the most common pain-related TMDs, such as myofascial pain with referral (sensitivity 0.86; specificity 0.98), arthralgia (sensitivity 0.89; specificity 0.98), headache attributed to TMD (sensitivity 0.89; specificity 0.87), and disc displacement without reduction with limited opening (sensitivity of 0.80 and specificity of 0.97) (13). The DC/TMD was validated for the Brazilian Portuguese language (14). The evaluator was trained and calibrated to use this tool.

#### Visual analogue scale (VAS)

The VAS is a linear scale, 10 cm in length, labeled at the two extremes with the boundaries of pain sensation: “no pain” at one end, and “worst pain imaginable” at the other end. The validity and reliability of these methods to determine pain intensity have been reported and confirmed in the literature (15, 16).

#### Bite dynamometer

The maximum bilateral molar bite force was assessed using a bite dynamometer, model DFM021115/200 (EMG System do Brasil, Sao Jose dos Campos, Brazil) with iron rods designed for the oral bite, protected by silicone material 15 mm thick. The device has a scale in kilograms of force (Kgf) with a reading capability from 0 to 200 kgf and was connected directly to one of the channels of the electromyographic acquisition module.

The bite dynamometer was used to evaluate the maximum bilateral molar bite force (MMBMax) during the proposed muscle fatigue protocol and in the bilateral molar bite task sustained at 50% of the maximum bite force (50% MMBs).

All volunteers received training prior to the test on the day of collection and were asked to bite the instrument stem with upper and lower molar teeth bilaterally.

#### Electromyography

The EMG 830C signal acquisition module (EMG System do Brasil, Sao Jose dos Campos, Brazil) was used to read the sEMG signals, with an impedance of 10 M*Ω*, analogue/digital converter, 16-bit resolution, sampling frequency of 2000Hz, fourth-order Butterworth filter (high-pass filter set at 20 Hz and low-pass filter set at 1000 Hz).

Four differential surface electrodes were used (self-adhesive, Ag/AgCl compounds, the electrodes had a conductive gel), with a fixed inter-electrode distance of 20 mm. The amplifier gain was 2000x, with a common rejection mode greater than 130 dB, input impedance of 10 G*Ω*, and background noise level less than 3 *μ*V.

The skin was cleaned with 70% alcohol before placement of the electrodes. Positioning followed the criteria proposed by Cram et al. (17), and a reference electrode (30 × 40 mm), consisting of a metal plate, was positioned on the manubrium of the sternum.

The data collection of the signals was carried out in a room with a controlled temperature maintained at 23° ± 2 °C and illuminated with incandescent lamps. The electromyographic signals collected were stored for later viewing and processing.

### Procedures

Initially, the consent form was signed by all volunteers who agreed to participate in the study. Subsequently, anthropometric data were collected (age, height, body mass), and the dominant chewing side established. Thereafter, volunteers were screened according to the DC/TMD and level of pain intensity on the face at rest, and the inclusion and exclusion criteria were verified. Finally, EMG evaluations of the masticatory muscles and maximum bilateral molar bite strength were performed in each volunteer.

During the EMG evaluation procedure and maximum bite force, the volunteers remained seated in a chair, with their back completely supported, feet parallel to the floor on a rubber mat, and arms supported over the anterior thighs (Frankfurt plane, parallel to the ground), as shown in Figure 2. The evaluations were performed for two repetitions of MMBMax (for evaluation of maximum bite force) and two repetitions of MMBS at 50% of MMBMax, which together represented the fatigue protocol, as detailed in Figure 3.

**Figure 2:**
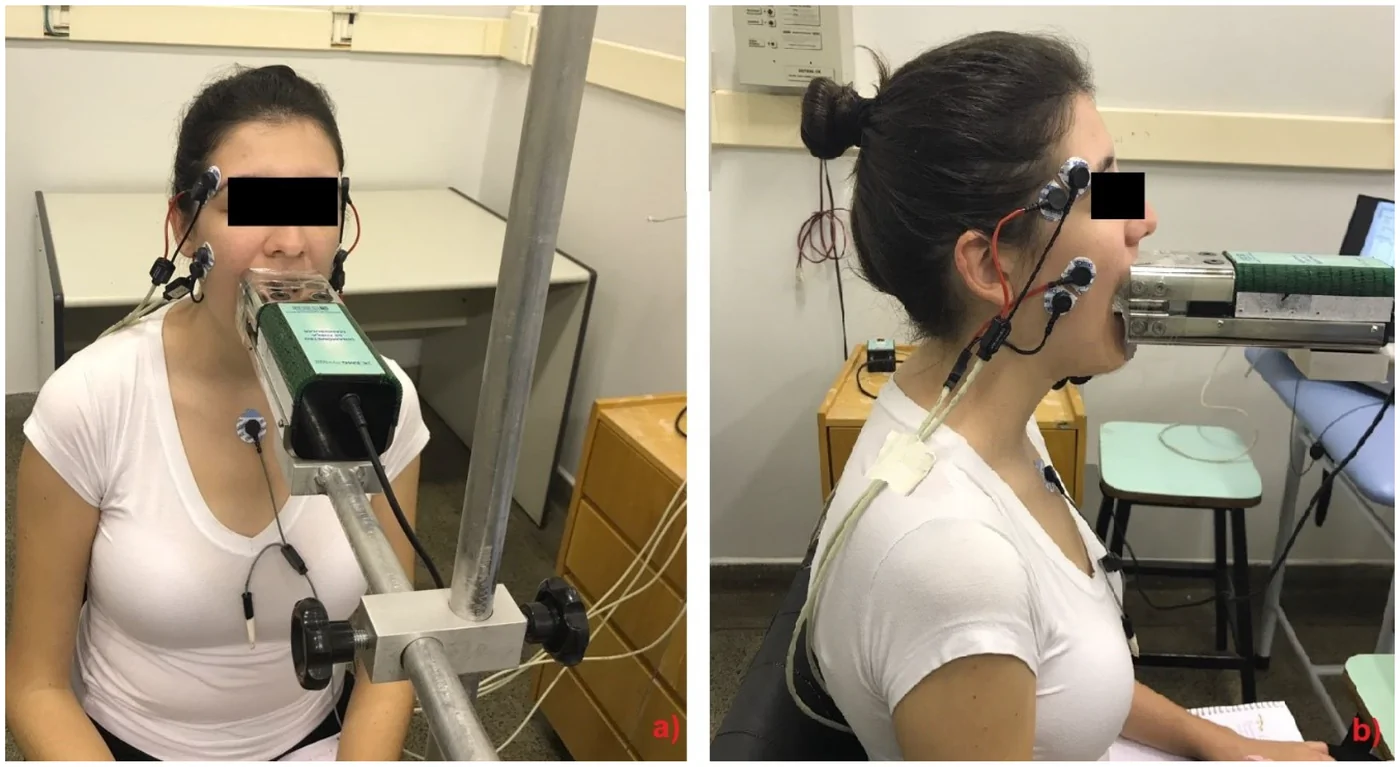
Positioning during the MMBMax **a)** anterior view and **b)** profile.

**Figure 3:**
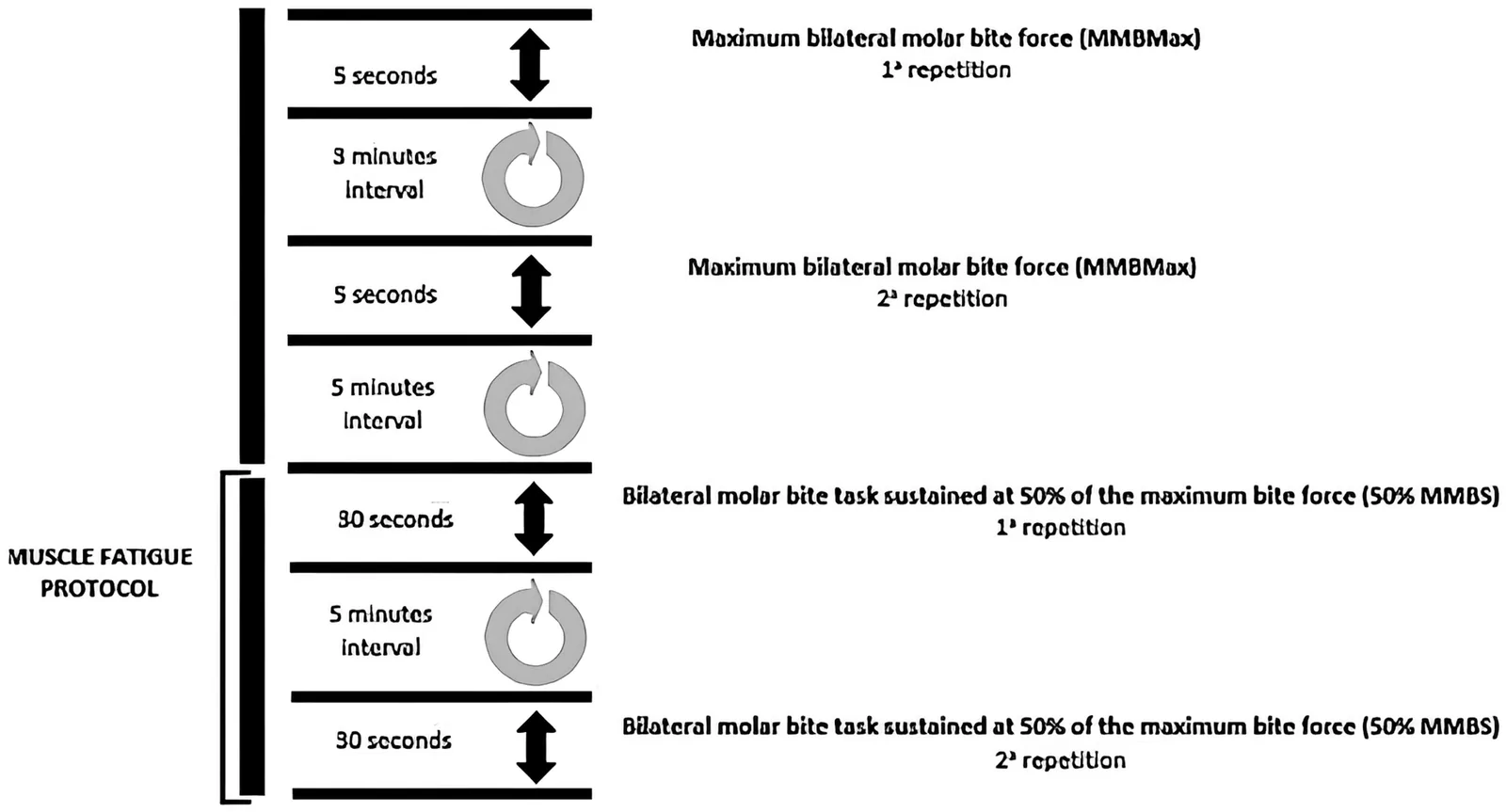
Description of the muscle fatigue protocol.

For the muscle fatigue protocol, during the MMBS task, the volunteers were asked to perform the bite on the dynamometer at a maintained strength of 50% of the maximum strength that each volunteer was able to perform in the MMBMax task. An average of 2 repetitions was used, with a 5-minute rest interval between repetitions. During the muscle fatigue protocol, the target force of 50% MMBMax was monitored in real-time using visual feedback provided by the acquisition software. Participants were instructed to maintain the force within an acceptable range of ±5% of the target value. During the 30-second task, deviations outside the ±5% target range occurred in a mean of 2.1 s per participant (range: 0–5.2 s). When such deviations occurred, the affected 2-second windows containing these periods were excluded from the regression analysis. All participants retained at least 12 windows for analysis. Both repetitions were analyzed separately, and the mean *β* coefficient was calculated for each participant.

Although the protocol allowed continuation in the presence of joint pain, participants could discontinue the task at any time if they experienced unacceptable discomfort or pain. Pain and discomfort were monitored throughout the protocol. No participant required interruption of the test. In addition, the evaluator provided verbal encouragement during the tasks.

### EMG signal processing and maximum bite force

The first and last 1 s of each EMG recording were eliminated to remove end-of-contraction artifacts from the analysis. This resulted in 28 s of usable signal per repetition, which was divided into 14 windows of 2 s to observe the linear regression line and angulation coefficient of the MF EMG and RMS EMG parameters. The RMS value was evaluated by the average of each window analyzed and, therefore, both the average RMS value and the MF EMG were evaluated over each of the 14 windows of 2 s.

For the final analysis, MF values (in Hz) and RMS values (in μV) were used without further normalization, as the primary outcome was the change in these parameters over time (regression slope) during the standardized 50% maximal bite force task. The normalized values were used solely for the pilot study sample size calculation.

### Statistical analysis

All research data were submitted to the normality test (Kolmogorov–Smirnov test) and presented normality. The data are described as mean, standard deviation, and 95% confidence interval. For intergroup comparisons of all research variables, the Student's t-test was used.

Simple linear regression analysis was performed for each volunteer in each group, considering the RMS and MF EMG parameters, to describe the relationship between the windows of the EMG parameters during the fatigue protocol in the MMBS. For this analysis the EMG of each masticatory muscle, with the coefficient of determination (r^2^) as the window time (14 windows of 2 s each), and the significance values of the analysis were jointly expressed (*p*-value). The angulation coefficient (*β*) was also calculated for each volunteer, in the follow-up, for comparison of the values between the groups, according to the fatigue protocol presented by Pitta (2015).

The analysis proceeded in two steps: 1) Individual level**:** a simple linear regression was calculated for each participant, with the 14 time windows as the independent variable and the EMG parameter (MF or RMS) as the dependent variable. This yielded an individual angulation coefficient (*β*) representing the rate of change over time for each muscle. 2**)** Group level**:** the individual *β* coefficients were then compared between the TMD and Control groups using the independent Student's t-test.

To determine whether the slope was significantly different from zero within each group, a one-sample t-test was performed on the individual *β* coefficients obtained from the linear regression for each participant. The mean *β* coefficient for each group was compared against zero. The results are reported as mean *β* ± SD and *p*-value from the one-sample t-test.

The primary outcome of this study was the intergroup comparison of the angulation coefficient (*β*) of the MF EMG linear regression for the four masticatory muscles. The RMS EMG parameters and intragroup regression analyses were considered secondary outcomes.

For the final analysis, the mean of the angulation coefficients (*β*) obtained from the two repetitions was calculated for each participant, providing a more stable estimate of the fatigue response.

Significance was set at 5% (*p* < 0.05). Given the multiple comparisons performed, the results should be interpreted with caution. We did not apply correction for multiple comparisons as these were pre-specified hypotheses based on previous literature, and the outcome measures are correlated. However, the risk of Type I error should be considered.

To assess the linearity assumption, we visually inspected scatter plots for each participant and performed the runs test for randomness. Non-linearity was observed in approximately 12% of cases. To evaluate the robustness of our results, we conducted a sensitivity analysis comparing the *β* coefficients obtained from linear regression with those from a non-parametric regression (Theil-Sen estimator). The correlation between the two methods was high (r > 0.88 for all muscles), confirming that the linear approach provides a reasonable approximation. Results from the linear regression are reported as the primary analysis.

Finally, an intergroup comparison of the mean angulation coefficient found in each group for the EMG parameters was performed. Data processing was performed using SPSS® software, version 17.0 (Chicago, IL, USA), and Graph Pad Prism 7.04. Significance was set at 5% (*p* < 0.05).

## Results

Table 1 shows the TMD classifications of all volunteers, diagnosed using the DC/TMD.

The groups also showed similarity related to dominance of the chewing side (X^2^ = 2.90; *p* = 0.14), since in the TMD group 15 volunteers presented dominance for chewing on the left side and 28 on the right side and, in the control group, 8 volunteers presented dominance on the left side and 35 on the right side.

Table 2 shows that the groups were similar with respect to BMI (*p* = 0.54) but differed significantly in age (*p* = 0.001) and maximum bite force (*p* < 0.001). The volunteers in the TMD group presented pain in the face both before and during the fatigue protocol, while the control group did not present pain.

**Table 2:** Intergroup (TMD and control group) comparisons of anthropometric data (age, BMI, pain intensity, and maximum bilateral molar bite force). Data are expressed as mean and standard deviation (*n* = 86).

Variable	TMD Group (Mean ± SD)	Control Group (Mean ± SD)	Statistical Test	*p*-value
Age (years)	28.72 ± 8.04	22.69 ± 6.94	t = 3.58	0.001*
BMI (kg/m^2^)	22.43 ± 2.51	22.74 ± 2.13	t = −0.61	0.54
Pain intensity at rest - VAS (cm)	3.13 ± 2.68	0 ± 0	NA	NA
Pain intensity after 1st 50% MMBS - VAS (cm)	5.76 ± 2.34	0 ± 0	NA	NA
Maximum bilateral molar bite force (Kgf)	30.85 ± 9.69	70.52 ± 11.13	t = −16.23	<0.001*

TMD, Temporomandibular Disorder; BMI, Body Mass Index; VAS, Visual Analogue Scale; MMBS, Maximum bilateral molar bite force; NA, Not applicable.

*Significant intergroup difference (Student's t-test).

Figures 4, 5 show the direction of the linear regression line of the masticatory muscles referring, respectively, to the MF EMG and RMS EMG values, considering the windows during the muscle fatigue protocol. It can be seen that the line has a downward direction for the MF EMG values for both groups, while for the RMS EMG of the masticatory muscles, the line has an upward direction for both groups. All angulation coefficients referring to the analysis of Figures 4, 5 are shown in Table 3, along with the corresponding t-values, degrees of freedom, and *p*-values from the one-sample t-test.

**Figure 4:**
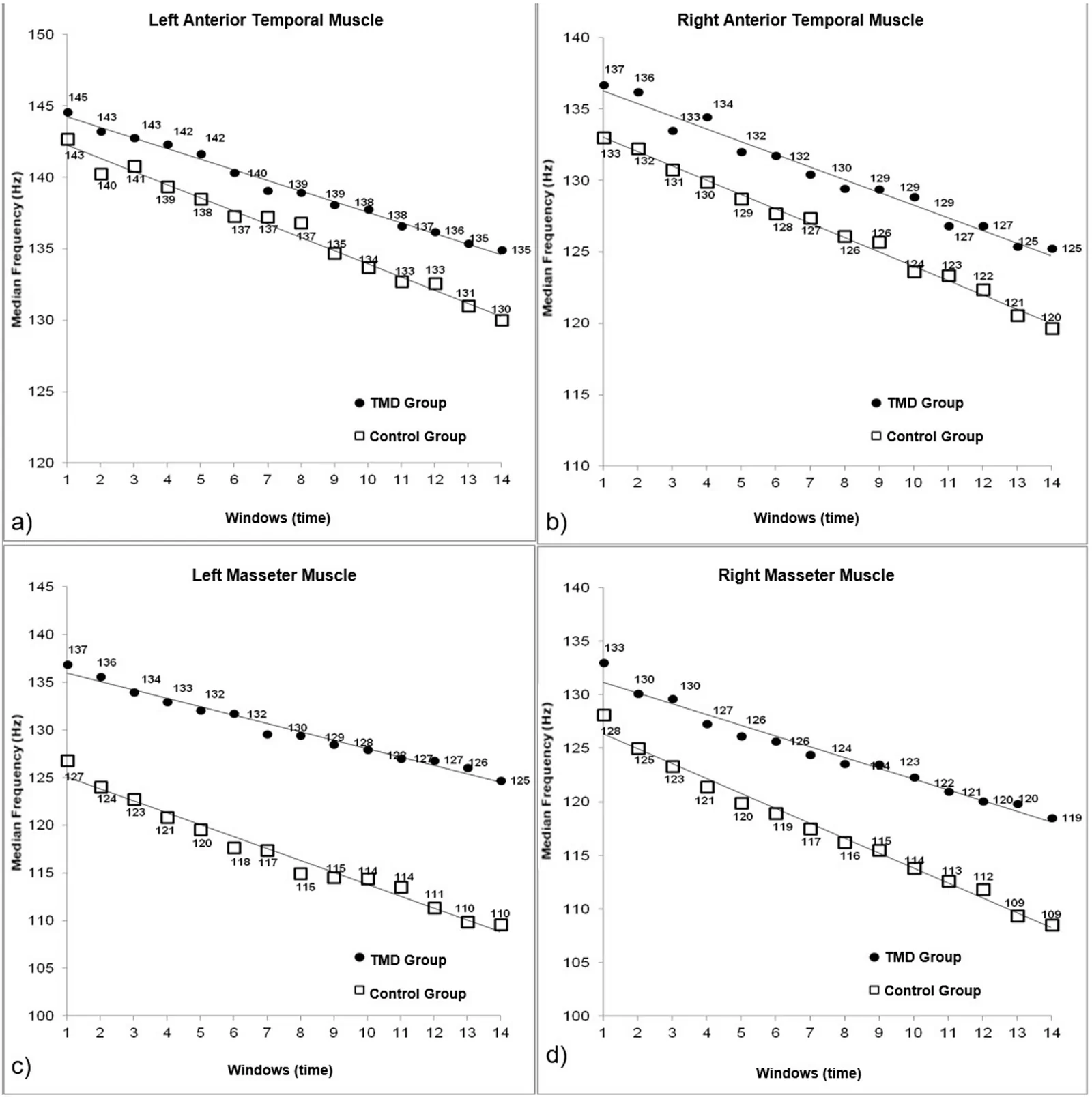
Linear regression lines of the median frequency (MF) EMG values during the muscle fatigue protocol, comparing TMD and Control groups. **(a)** Left anterior temporalis muscle; **(b)** right anterior temporalis muscle; **(c)** left masseter muscle; **(d)** right masseter muscle. Black circles represent the TMD Group and white squares represent the Control Group. The x-axis represents time windows (1–14) and the y-axis represents median frequency (Hz).

**Figure 5:**
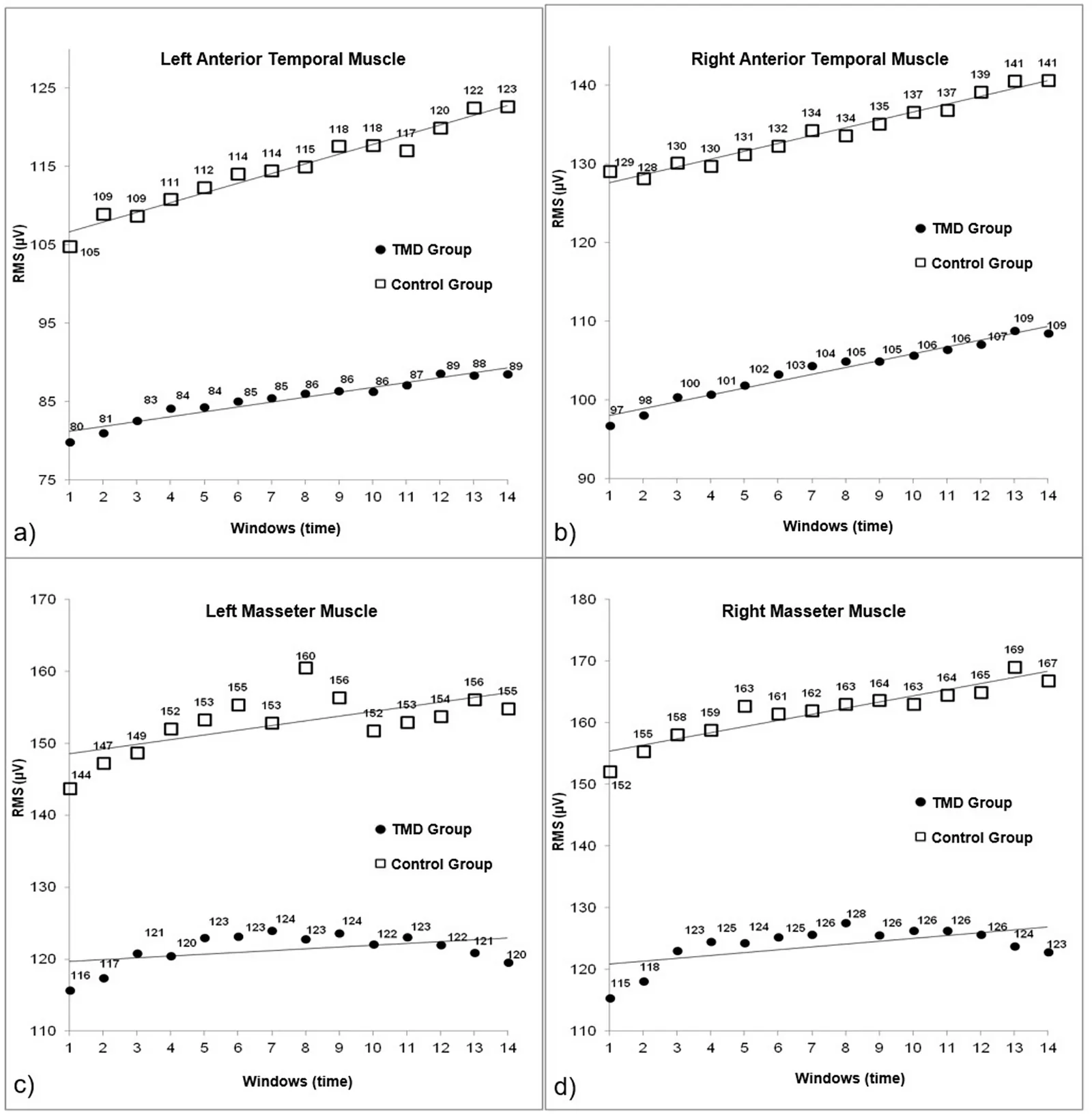
Linear regression lines of the root mean square (RMS) EMG values during the muscle fatigue protocol, comparing TMD and Control groups. **(a)** Left anterior temporalis muscle; **(b)** right anterior temporalis muscle; **(c)** left masseter muscle; **(d)** right masseter muscle. Black circles represent the TMD Group and white squares represent the Control Group. The x-axis represents time windows (1-14) and the y-axis represents RMS (μV).

**Table 3:** Intra and intergroup (TMD and Control group) analysis of the angulation coefficients (*β*) of the linear regression line for MF EMG and RMS EMG parameters during the muscle fatigue protocol. Data are expressed as mean and standard deviation (*n* = 43 per group).

EMG Parameter/Muscle	Group	*β* (Mean ± SD)	Intragroup Analysis (One-sample t-test against zero)	Intergroup Analysis (Student's t-test)
MF EMG - Left Anterior Temporalis	TMD	−0.09 ± 0.08	t = −7.38; df = 42; *p* < 0.001	t = 0.89; *p* = 0.37
	Control	−0.11 ± 0.07	t = −10.30; df = 42; *p* < 0.001	
MF EMG - Right Anterior Temporalis	TMD	−0.11 ± 0.07	t = −10.30; df = 42; *p* < 0.001	t = 0.89; *p* = 0.37
	Control	−0.13 ± 0.07	t = −12.17; df = 42; *p* < 0.001	
MF EMG - Left Masseter	TMD	−0.11 ± 0.08	t = −9.01; df = 42; *p* < 0.001	t = 2.53; *p* = 0.01
	Control	−0.16 ± 0.08	t = −13.10; df = 42; *p* < 0.001	
MF EMG - Right Masseter	TMD	−0.13 ± 0.09	t = −9.47; df = 42; *p* < 0.001	t = 2.45; *p* = 0.01
	Control	−0.18 ± 0.09	t = −13.10; df = 42; *p* < 0.001	
RMS EMG - Left Anterior Temporalis	TMD	0.08 ± 0.08	t = 6.55; df = 42; *p* < 0.001	t = −2.48; *p* = 0.01
	Control	0.16 ± 0.19	t = 5.52; df = 42; *p* < 0.001	
RMS EMG - Right Anterior Temporalis	TMD	0.11 ± 0.10	t = 7.21; df = 42; *p* < 0.001	t = −0.37; *p* = 0.70
	Control	0.12 ± 0.26	t = 3.03; df = 42; *p* = 0.004	
RMS EMG - Left Masseter	TMD	0.03 ± 0.25	t = 0.79; df = 42; *p* = 0.43	t = −0.88; *p* = 0.38
	Control	0.08 ± 0.25	t = 2.10; df = 42; *p* = 0.04	
RMS EMG - Right Masseter	TMD	0.05 ± 0.23	t = 1.43; df = 42; *p* = 0.16	t = −1.30; *p* = 0.19
	Control	0.12 ± 0.26	t = 3.03; df = 42; *p* = 0.004	

MF, Median frequency; EMG, Electromyography; RMS, Root Mean Square; TMD, Temporomandibular Disorder; β, angulation coefficient of the linear regression line; df,  degrees of freedom.

Intragroup significance indicates whether the mean *β* coefficient differs significantly from zero (one-sample t-test). Intergroup significance indicates a significant difference between β coefficients of TMD and Control groups (Student's t-test).

Table 3 shows the intragroup analysis using one-sample t-tests on the individual *β* coefficients against zero. The one-sample t-test revealed that the mean *β* coefficients were significantly different from zero for MF EMG in all muscles in both groups (*p* < 0.001 for all), indicating a significant decline in MF over time during the fatigue protocol. For RMS EMG, significant intragroup differences were found in the control group for all muscles (*p* < 0.001 for the anterior temporalis muscles and *p* = 0.004 for the masseter muscles) and in the TMD group for both anterior temporalis muscles (*p* < 0.001 for both), but not for the masseter muscles in the TMD group (left masseter: *p* = 0.43; right masseter: *p* = 0.16).

The intergroup comparison of *β* coefficients revealed significantly greater negative *β* values for the Control Group compared to the TMD Group in the MF of the right masseter (*p* = 0.01) and left masseter (*p* = 0.01) muscles, indicating a steeper decline in MF in the Control Group. A significantly greater positive *β* value was observed for the Control Group in the RMS of the left anterior temporalis muscle (*p* = 0.01), indicating a steeper increase in RMS in the Control Group. No significant intergroup differences were found for the other muscles or parameters.

## Discussion

TThe present study aimed to compare the EMG parameters (MF and RMS) of the masseter and anterior temporalis muscles during a long-duration (30-second) fatigue protocol involving a sustained bilateral submaximal molar bite task in women with myogenic TMD and asymptomatic women. Our findings demonstrated that the long-duration submaximal bite task produced signs of muscle fatigue in both groups; however, signs of fatigue were significantly more pronounced in the Control Group, specifically in the masseter muscles (MF decline) and the left anterior temporalis muscle (RMS increase).

An important consideration in interpreting our results is that, although the task was standardized at 50% of each participant's maximum bite force, the absolute forces differed substantially between groups (Control: 70.52 Kgf vs. TMD: 30.85 Kgf). This difference could be interpreted as a possible contributing factor to the more pronounced signs of fatigue observed in the Control Group. One possible interpretation is that individuals with TMD may be relatively protected from fatigue by their reduced force-generating capacity, as the absolute demands of the task are lower. However, this hypothesis requires direct testing in future studies. The consistently higher MF EMG values observed in the TMD group may be attributed to the significantly lower intensity of the contractions, as the Median Frequency normally decreases with increasing intensity of contraction.

The results of the present study partially corroborate and partially diverge from previous findings in the literature. Pitta et al. (2015) found significant angulation coefficients for the MF EMG values for the anterior temporal and masseter muscles in both groups but no significant differences between the groups (TMD and asymptomatic). However, significantly greater electrical activity (RMS EMG) was found in the asymptomatic group compared to the TMD group for the anterior temporalis muscle. These findings corroborate with the present study, however, there were also no more significant coefficients of MF EMG angulation of the masticatory muscles in the control group compared to the myogenic TMD group during the fatigue protocol. It is possible that this lack of difference is because a force transducer/bite dynamometer was not used in Pitta et al. (2015), and because the authors performed a short muscle fatigue protocol. These factors may have attenuated the effects of MF on the masticatory muscles between groups.

Xu et al. (2017) evaluated two groups, the TMD group and asymptomatic group, in a fatigue protocol for the maximum tolerated time at 30% of the maximum bite force, using a bite dynamometer. Significantly higher normalized RMS EMG values were observed in all muscles evaluated in the TMD group when compared to the asymptomatic group. In addition, for the anterior temporal muscle, a significant increase in normalized RMS EMG values from the beginning to the end of the task was reported only for the bite side (due to a unilateral evaluation) for the asymptomatic group, validating the findings of the present research. Furthermore, there was a significant reduction in the MF EMG values of the masseter and anterior temporal muscles on the bite side for both groups, but no significant intergroup difference. These results related to the MF EMG could be different from the present study due to the use of a unilateral molar bite dynamometer, which may have masked the signs of EMG fatigue in the MF, since the Central Nervous System activates the masticatory muscles bilaterally and not unilaterally during the bite (5).

The greater signs of fatigue observed in the Control Group may be explained by several factors. First, the Control Group demonstrated significantly higher maximum bite force, meaning that at 50% of their maximum, they were generating substantially higher absolute forces than the TMD Group. This higher absolute force likely resulted in greater metabolic demand and more pronounced muscle fatigue (8). Second, individuals with TMD may employ protective motor strategies to avoid pain, which could limit the actual force production and consequently reduce the observable signs of fatigue (20). Third, it is possible that chronic pain conditions result in neuromuscular adaptations that alter the fatigue response (21).

Despite the similarity found in the analysis of the side of masticatory preference between the groups of this research, a significantly higher angle of the coefficient of the RMS EMG parameter was observed in the left temporal muscle of the control group during the fatigue protocol. It is possible this increase in the RMS EMG specifically on the left side of the anterior temporal muscle may be due to the greater preference for chewing on the opposite side found in the group, since electrical activity in the left muscle increases throughout the protocol in an attempt to maintain the submaximal strength level for 30 s, while the right muscle does not show evident signs of fatigue because it is more adapted. On the other hand, it is also possible that the difference between the left and right results of the TMD group occurred due to chance. This probably accounts for the significant difference between the left masseter muscles of the TMD group and controls, rather than it being because this was the non-dominant side. Therefore, as the present research cannot fully explain the EMG difference found between the muscular sides, the results must be interpreted with caution and studied in future research.

### Strengths and limitations

This study was conducted by a group of trained therapists. A differential of this study was the use of a bilateral dynamometer to perform a submaximal bite in a long fatigue protocol. Although the study was carefully carried out, some limitations need to be recognised such as: a) absence of an evaluation of the maximum bite force after the proposed fatigue protocol, since this would complement the evident EMG fatigue findings in the control group; b) absence of an evaluation of the facial pattern of the volunteers in this research, since individual differences can contribute to altering the maximum bite force and electrical activity of the masticatory muscles during bite tasks; c) the results obtained can be applied only to young women and during a bite on a 15 mm thick bilateral molar dynamometer as used in this research; d) a limitation of this study is the absence of simultaneous recording of bite force and EMG signals, which would have allowed for more precise correlation between force output and electrical activity during the task. Future studies could explore integrated force-EMG recording systems to address this limitation; e) we do not have information about the length of time of the chronic pain and the type of food the volunteer preferred, or the degree and type of bruxism; and f) caution should be taken in statistical interpretation because linear regression may not be the most precise model when the data are not strictly linear (as observed in some cases). Future studies might explore non-linear models (e.g., polynomial or exponential regression) to better characterize fatigue trajectories.

### Research and clinical applications

Based on the findings of the present study, EMG appears to be a tool capable of evaluating fatigue in individuals with TMD. Future research should continue investigating fatigue protocols in TMD populations using EMG parameters. The evaluation of bite force after the fatigue protocol is also recommended for both asymptomatic and TMD groups.

It is established in the literature that individuals with TMD often perform submaximal bite activities during daily functions such as chewing, as pain limits their ability to generate maximum bite force (18, 19). Our findings demonstrate that during submaximal bite, women with myogenic TMD present significant changes in the electrical activity of the masseter and left temporal muscles. We hypothesize that therapeutic interventions targeting these muscles could potentially yield promising clinical outcomes; however, this hypothesis requires direct investigation in controlled interventional studies.

## Conclusion

It is concluded that the research hypothesis was rejected since there were significantly greater differences in some muscles of the control group compared to the myogenic TMD group, that is, a greater coefficient of MF EMG angulation for the masseter muscles (downward direction) and higher RMS EMG angulation coefficient for the left temporal muscle (upward direction) in the control group during the proposed muscle fatigue protocol. However, as expected, a long submaximal fatigue protocol was able to produce fatigue in both groups (asymptomatic and TMD).

## Data Availability

The original contributions presented in the study are included in the article/Supplementary Material, further inquiries can be directed to the corresponding author.
